# Asymmetric spillover between economic policy uncertainty and exchange rate volatility: A global network connectedness perspective

**DOI:** 10.1371/journal.pone.0279729

**Published:** 2023-01-26

**Authors:** Panpan Wang, Yishi Li, Xiaoxing Liu

**Affiliations:** 1 School of Economics and Management, Southeast University, Nanjing, Jiangsu Province, China; 2 College of Economics and Management, Zhejiang University of Water Resources and Electric Power, Hangzhou, Zhejiang Province, China; Universidad de Salamanca, SPAIN

## Abstract

This study employs the network connectedness approach to examine the risk spillover between the economic policy uncertainty (EPU) and exchange rate volatility (ERV) of 21 countries. Using monthly data from January 1997 to August 2022, we find that the spillover effect of ERV on EPU is greater than that of the inverse. In addition, the spillover effect of EPU on ERV is mainly concentrated in the foreign exchange markets of developing countries. This finding indicates that the foreign exchange markets of developing countries are more susceptible to shocks of global economic risk, and the spreading of risk contagion between EPU and ERV mainly follows the pathway “increase in global ERV → rising global EPU → further intensified volatility in the foreign exchange markets of developing countries.” A rolling-window analysis shows that the spillover between global EPU and ERV is time-varying. The cross-market spillovers between EPU and ERV in the post-crisis period continued to rise and further increased sharply after the outbreak of the COVID-19 pandemic.

## 1. Introduction

In addition to affecting economic activities such as investment and consumption [1–3], economic policy uncertainty (EPU) is an important driver of asset price dynamics [4–6]. Following the 2008 global financial crisis (GFC), the frequent adjustment of economic policies in various countries has led to a significant increase in global EPU. The impact of EPU on exchange rate dynamics has attracted the attention of concerned parties. From a macro perspective, the rise in EPU indirectly affects exchange rates by affecting the state of the economy [7]. From a micro perspective, the existence of EPU leads to investors’ heterogeneous expectations on the economic fundamentals that determine exchange rates, thereby triggering exchange rate volatility (ERV) [8–10].

Previous studies have mainly focused on the impact of EPU on ERV, generally finding that EPU has a cross-border spillover effect on ERV. Based on the structure of cross-border spillovers, existing studies can be divided into three categories: investigating the impact of domestic and foreign EPU on the ERV of domestic markets [11–14]; studying the impact of the EPU of the core country (i.e., the United States) on the ERV of other countries [15]; and exploring the impact of the relative EPU of two countries on bilateral ERV [7, 8, 16]. From the results in relevant studies, Krol [11] shows that a rise in both domestic and US EPU increases domestic ERV based on a study of ten developed and developing economies. Al-Yahyaee et al. [13] find the same in eight developed and developing economies, and Abid and Rault [17] also find the same in eight emerging market economies. Moreover, Abid and Rault [17] further show that the contribution of US EPU to ERV fluctuations in emerging market economies even exceeds that of home EPU, implying that the US, as the most important developed country, has extensive spillover effects from its EPU to the foreign exchange markets of countries around the world, especially emerging markets and developing countries. In country-specific studies, related findings also show that ERV in developing economies is not only influenced by their own EPU but is also subject to the significant influence of EPU in developed economies. For example, for the ERV of the Chinese RMB, studies have shown that it is significantly influenced not only by Chinese EPU but also by US, European and Japanese EPU [12, 18–20]; for the ERV of the Mexican peso, it is also influenced by both domestic EPU and foreign EPU [21]. Regarding the ERV of currencies of developed economies, related studies have found that they are mainly influenced by EPU within developed countries. For example, Bartsch [22] finds that the volatility of the GBP/USD exchange rate is influenced by the UK and US EPU, and Nilavongse et al. [14] further suggest that the UK EPU is the main source of the ERV of the GBP.

Furthermore, studies have shown that the spillover effects of EPU on ERV are further intensified during crisis periods or in economic recessions. For instance, Krol [11] finds that the positive impact of EPU on ERV is further enhanced in times of economic underperformance. Kido [15] shows that the spillover effects of US EPU on the ERV of eight developed and developing countries were further intensified during the two recessions after 9/11 in 2001 and after the GFC in 2008. Bush and Noria [21] also show that the amplifying effect of Mexico’s domestic economic uncertainty on its ERV is further enhanced during the economic recession. Al-Yahyaee et al. [13] find that the linkage between EPU and the foreign exchange market was further strengthened during the 2008–2009 GFC. Li and Zhong [19] suggest that the spillover effects of global EPU shocks on China’s financial conditions index (which includes a component of the RMB exchange rate) are concentrated in two crisis periods, one during the 2008–2009 GFC and the other during the 2012–2013 European sovereign debt crisis. Wang et al. [10] show that the impact of US EPU on the ERV of the RMB is further enhanced following the outbreak of Sino-US trade friction in 2018. Nilavongse et al. [14] find that the surge in domestic EPU in the UK after the Brexit referendum was the significant cause of the large fluctuation of the GBP.

Although there have been rich studies on the spillover effects of EPU on ERV, several gaps still remain. First, such studies have focused primarily on the spillover effect of EPU on ERV and have not accounted for the reverse. ERV reacts on economic fundamentals, thereby affecting the formulation of economic policies and increasing policy uncertainty. Studies have shown that ERV is an important factor affecting the formulation and adjustment of a country’s economic policies, such as monetary and trade policies [23, 24], while the expansion of ERV further deepens the uncertainty of economic policies. Second, the cross-border and cross-market contagion effects between EPU and ERV tend to form a complex risk transmission network at the global level. To our knowledge, no study has yet explored the cross-border and cross-market spillover effects between EPU and ERV under a network framework; the exploration of the complex spillover relationships between the two factors and the identification of the dynamic changes in the spillover network are insufficient.

This study contributes to the literature by applying a network topology method to analyze the cross-border and cross-market risk spillover effects between EPU and ERV. The contributions of this study are twofold: (i) we consider the bidirectional spillover effects between EPU and ERV, and (ii) we establish a global spillover network between EPU and ERV to demonstrate that EPU and exchange rates are subject not only to cross-border spillover in their respective markets but also to cross-market and cross-border spillover effects on one another. Through the network connectedness approach, we can systematically analyze the cross-border and cross-market spillover between EPU and ERV, as well as the risk contagion pathways. Furthermore, a rolling-window analysis is also used to identify the dynamic evolution of such spillover effects.

As a result, we show that the spillover effect of ERV to EPU is stronger than the spillover effect in the opposite direction, while the spillover of EPU to ERV is largely concentrated in developing countries, i.e., the foreign exchange markets of developing countries are more vulnerable to global economic risks. In addition, cross-market spillovers between global EPU and ERV continued to rise after the 2008 GFC and have further increased rapidly and substantially since the outbreak of the COVID-19 pandemic in 2020. Our findings are conducive to correctly evaluating the position of each country’s EPU and ERV in the global risk spillover network, as well as a better understanding of how the policy shock of a single country spreads to the global foreign exchange market through network connectedness. The findings also provide a new tool for predicting ERV risk so that risk resonances caused by cross-border and cross-market risk contagions may be better prevented.

The rest of the paper is structured as follows. Section 2 explains the methodology and the data. The empirical results are discussed in Section 3. Section 4 concludes.

## 2. Methodology and data

### 2.1 Methodology

This study employs Diebold and Yilmaz’s [25] spillover measures based on generalized forecast error variance decomposition (FEVD) to construct the spillover network for the EPU and ERV of various countries around the world. The use of the Diebold and Yilmaz [25] spillover analytical framework can help systematically examine the cross-country and cross-market spillover features between global EPU and ERV and the structure of the risk transmission path and thus scientifically evaluate the position of each country’s economic policy and foreign exchange market in the global risk transmission chain. This would contribute to our understanding of how individual or partial countries’ policy shocks spread to the global foreign exchange market through network linkages and facilitate better prevention of global risk resonance triggered by cross-country and cross-market contagion. In applications of Diebold and Yilmaz [25] spillover analytical framework, we base the spillover assessment on a high-dimensional VAR model. To ensure that VAR is estimable in high dimensions, we use the sparse-group lasso approach (short for "least absolute shrinkage and selection operator"), which facilitates high dimensionality by selecting and shrinking in optimal ways [26, 27]. Specifically, the construction of the network spillover index involves three steps. First, the sparse-group lasso VAR model is used to construct an N-dimensional p-order VAR model; next, generalized FEVD is performed; and finally, the spillover indices are calculated.

#### 2.1.1 Sparse-group lasso VAR

The N-dimensional p-order *VAR*_*N*_(*p*) model is constructed, $y_{t}=v+\sum_{i=1}^{p}\Phi_{i}y_{t-i}+\varepsilon_{t}$, where *y*_*t*_ is an N ×1 vector of endogenous variables, *v* is an N ×1 vector of constants, *ε*_*t*_ is an N × 1 i.i.d. random vector of disturbance, and Φ_*i*_ denotes the parameter matrix. The VAR model is over-parameterized as the number of parameters increases quadratically with the number of variables. Thus, with the increase in dimensions, the model risks a “curse of dimensionality.” To resolve the problem of over-parameterization, the structured regularization model, sparse-group lasso VAR, is used in this study [26]. The model embeds both the group-wise sparsity on the coefficients and within-group sparsity of the lagged values of the variables. The model can be expressed as follows.

$$\underset{v,\Phi }{\min}\sum_{t=1}^{T}{\left\|y_{t}-v-\sum_{i=1}^{p}\Phi_{i}y_{t-i}\right\|}_{F}^{2}+\lambda ((1-\alpha )N\sum_{i=1}^{p}{\left\|\Phi_{i}\right\|}_{F}+\alpha {\left\|\Phi \right\|}_{1})$$ (1)

where N denotes the dimensionality of the VAR model, p denotes the maximum lag order, and T denotes the length of the time series. ${\left\|A\right\|}_{F}=\sqrt{\sum_{ij}a_{i,j}^{2}}$ is the *F*-norm of matrix A. ‖*A*‖_1_ = ∑_*ij*_|*a*_*i*,*j*_| is the ℓ1-norm of matrix A. *λ* represents the regularized inter-group penalty term, *λ*≥0. *α* is the penalty term that controls the within-group sparsity, *α*∈[0,1]. When *α* = 0, the parameter estimation is the group-wise sparsity model; when *α* = 1, parameter estimation is assessed with the basic lasso VAR model.

Referring to the practice of Nicholson et al. [28], it is set that *α* = 1/(*N*+1). Rolling cross-validation is employed to estimate the optimal penalty term *λ*. The time series of length *T* is divided into three equal periods, where *T*1 = ⌊*T*/3⌋, *T*2 = ⌊2*T*/3⌋. The data from 1 to *T*1 are used to initialize the covariate matrix, the data from *T*1 to *T*2 are used for parameter selection, and the data from *T*2 to *T* are used for forecast evaluation. Finally, the optimal *λ* is selected according to the minimized 1-step forward prediction mean-square forecast error (MSFE) to thereby obtain the model’s estimation results. The MSFE is calculated as follows.


$$MSFE(\lambda_{i})=\frac{1}{\left(T_{2}-T_{1}\right)}\sum_{t=T_{1}}^{T_{2}-1}{\left\|{\hat{y}}_{t+1}^{\lambda^{i}}-y_{t+1}\right\|}_{F}^{2}$$ (2)

#### 2.1.2 Generalized FEVD

Based on the generalized FEVD method proposed by Pesaran and Shin [29], the VAR model is converted into the vector moving average representation: $Y_{t}=\sum_{j=1}^{\infty }\Psi_{j}\varepsilon_{t-j}$, where Ψ_*j*_(*j* = 1,2,3,⋯) satisfies the recursive expression $\Psi_{j}=\Phi_{1}\Psi_{j-1}+\Phi_{2}\Psi_{j-2}+\cdots +\Phi_{p}\Psi_{j-p};\;\Psi_{0}$ is the identity matrix of *N*×*N*; and when *j*<0, Ψ_*j*_ = 0. Thus, the *N*×*N* generalized variance decomposition matrix (Θ_*t*_) is obtained, and each element in the matrix is obtained using the following formula.

$$\theta_{jk}^{H}=\frac{\sigma_{kk}^{-1}\sum_{h=0}^{H-1}{\left({e^{\prime }}_{j}\Psi_{h}\Sigma e_{k}\right)}^{2}}{\sum_{h=0}^{H-1}\left({e^{\prime }}_{j}\Psi_{h}\sum {\Psi^{\prime }}_{h}e_{j}\right)}$$ (3)

where $\theta_{jk}^{H}$ is the element in the *j*-row and *k*-column of the *H*-step variance decomposition matrix, indicating the proportion of the total predicted variance of the *j*th variable that is generated from the *k*th variable. Σ is the covariance matrix of the disturbance vector *ε*. *σ*_*kk*_ is the *k*th diagonal element of matrix Σ, indicating the variance of the *k*th disturbance term. *e*_*j*_ and *e*_*k*_, which are the selection vectors, represent the vectors in the *j*th and *k*th columns of the identity matrix, respectively. Notably, the sum of the contributions of the prediction errors of generalized variance decomposition is usually not equal to 1. To be consistent with the economic significance of the traditional variance decomposition method (to ensure that the sum is equal to 1), the generalized variance decomposition matrix is normalized by the sum of the rows and transformed into a normalized generalized variance decomposition matrix. Each element in the normalized matrix is expressed as follows.

$${\tilde{\theta }}_{jk}^{H}=\frac{\theta_{jk}^{H}}{\sum_{k=1}^{N}\theta_{jk}^{H}}$$ (4)

where ${\tilde{\theta }}_{jk}^{H}$ is the shock from individual *k* on the variance of the *H*-step generalized forecast error of individual *j*, indicating the spillover effect of *k* on *j*.

#### 2.1.3 Construction of the spillover index

Based on the normalized generalized variance decomposition matrix and referring to the method proposed by Diebold and Yilmaz [25], the following network spillover indices are constructed.

(1) Total spillover (TS). The total spillover index is calculated by dividing the sum of all non-diagonal elements of the normalized generalized variance decomposition matrix (${\tilde{\Theta }}_{H}$) by the number of elements. The total spillover index can be used to measure the overall spillover between global EPU and ERV. The calculation is as follows.
$$TS=\frac{1}{N}\sum_{j,k=1,j\neq k}^{N}{\tilde{\theta }}_{jk}^{H}$$ (5)
(2) Directional spillover. The directional spillover indices allow us to describe the external spillover effects both from a given element in the network and the spillover shocks the element received. The calculations are as follows.
$$Out(j)=\sum_{k=1,j\neq k}^{N}{\tilde{\theta }}_{kj}^{H}$$ (6)
$$In(j)=\sum_{k=1,j\neq k}^{N}{\tilde{\theta }}_{jk}^{H}$$ (7)
  where “Out” is defined as the outward spillover index, which represents the total spillover of individual *j* on all other individual elements. A larger “out” index indicates a greater outward spillover effect of element *j*. “In” is defined as the inward spillover index, which represents the total spillover that individual *j* received from all other individual elements. A larger “in” index indicates greater external shocks received by element *j*.
(3) Market-specific spillover. To evaluate the spillover of a given market’s individual element on another type of market or the spillover that a given market’s individual element receives from another type of market, the following indices are constructed.
$$TOTO(j)=\sum_{k=1,k\notin m}^{{N-N}_{m}}{\tilde{\theta }}_{kj}^{H}$$ (8)
$$TIFO(j)=\sum_{k=1,k\notin m}^{{N-N}_{m}}{\tilde{\theta }}_{jk}^{H}$$ (9)
  where *N*_*m*_ denotes the number of individual elements in market *m*. The total-out-to-others (TOTO) index reflects the sum of the spillovers formed by *j* (from market *m*) on *k* (from another type of market). The total-in-from-others (TIFO) index reflects the sum of the spillovers that *j* (from market *m*) receives from *k* (from another type of market).
(4) Sector spillover. The sector spillover index is used to measure the spillover from one market to another. The calculation is as follows.
$$S_{m\to n}=\sum_{j=1,j\in m}^{N_{m}}\sum_{k=1,k\in n}^{N_{n}}{\tilde{\theta }}_{kj}^{H}$$ (10)
  where *S*_*m*→*n*_ denotes the spillover from market *m* to market *n*. *N*_*m*_ and *N*_*n*_ denote the number of individual elements in markets *m* and *n*, respectively. A larger *S*_*m*→*n*_ indicates a stronger influence of market *m* on *n*.

### 2.2 Data

This study aims to construct a spillover network between EPU and ERV in countries around the world, so we seek to select EPU and exchange rate data from as many countries as possible as our sample. Our EPU data are derived from Baker et al. [1], who construct and publish monthly EPU indices for major countries worldwide based on news information in newspapers. The EPU index data constructed and published by Baker et al. [1] are available at http://www.policyuncertainty.com. However, the number of countries included in the EPU database of Baker et al. [1] is somewhat limited, and we obtain monthly EPU index data for a maximum of 21 major countries in their database, including 14 developed and 7 developing countries. The 14 developed countries include Australia, Canada, France, Germany, Greece, Ireland, Italy, Japan, South Korea, Netherlands, Spain, Sweden, the United Kingdom and the United States. The 7 developing countries include Brazil, Chile, China, Colombia, India, Mexico and Russia. The corresponding exchange rate data for those countries are obtained from the nominal effective exchange rate database published by the Bank of International Settlement, and the frequency of the raw exchange rate data is daily. The sample ranges from January 1997 to August 2022. The data are processed as follows. First, the EPU index of each country is processed using the logarithmic difference to obtain the rate of change of EPU. Next, the log-return rate of each country’s daily exchange rate is calculated. Then, the monthly-compounded standard deviation of the exchange rate return is calculated to yield the ERV index. It is worth mentioning that our raw exchange rate data are daily-frequency and raw EPU data are monthly-frequency, while modeling high-frequency daily exchange rate data could have the problem of data asynchrony [30]. However, we process the raw data by calculating the monthly standard deviation of daily-frequency exchange rate returns for each country to obtain the monthly-frequency ERV indicator and then perform VAR modeling on the monthly-frequency ERV and EPU. This lowering the frequency of the data from daily to monthly helps to address the potential problem of asynchronization [31, 32].

## 3. Results

### 3.1 Analysis of spillover indices of global EPU and ERV

Based on the Akaike information criterion, the optimal lag order of the lasso VAR model is set to be *p* = 2; the forecast period of the generalized FEVD is set to *H* = 3. Panel A of Table 1 shows various types of network spillover indices between EPU and ERV. The means of the “in” and “out” indices of the ERV are 76.24 and 80.55, respectively, ranking first among the indices and indicating that in the spillover network of global EPU and ERV, the foreign exchange market is both the main exporter and receiver of risk. The foreign exchange market is both most likely to transmit shocks externally and the most vulnerable to external shocks. However, the “in” and “out” indices not only included the cross-market spillovers between EPU and ERV but also the spillovers within the corresponding markets. To measure the cross-market spillovers between EPU and ERV, the TIFO and TOTO indices are calculated. A comparison between the two indices reveals that the average spillover effect of ERV on EPU is 8.18, which is approximately 2.1 times the average spillover effect of EPU on ERV (3.88).

**Table 1:** Spillover indices between EPU and ERV.

**Panel A. Network spillover indices**												
	**In**			**Out**			**TIFO**			**TOTO**		
	**Min**	**Mean**	**Max**	**Min**	**Mean**	**Max**	**Min**	**Mean**	**Max**	**Min**	**Mean**	**Max**
**ERV**	49.27	76.24	87.24	22.99	80.55	120.20	1.22	3.88	8.52	2.80	8.18	16.88
**EPU**	20.45	50.02	70.78	8.87	45.72	99.74	1.74	8.18	16.42	0.55	3.88	7.23
**Panel B. Sector spillover**												
	**Sector spillover**						**Sector spillover as % of all spillover**					
**From\To**			**ERV**			**EPU**			**ERV**			**EPU**
**ERV**			1519.70			171.81			57.31			6.48
**EPU**			81.43			878.71			3.07			33.14

Note: (i) This table reports various types of spillover indices between economic policy uncertainty and exchange rate volatility. (ii) ERV represents exchange rate volatility, and EPU represents economic policy uncertainty. (iii) "In" and "out" are the inward and outward spillover indices, respectively. They represent the total spillover of all other individuals on a single individual and the total spillover of a single individual on all other individuals, respectively. The "In" and "Out" indices can be calculated using Eq (7) and Eq (6), respectively. (iv) TIFO and TOTO are market-specific spillover indices, and the two denote total-in-from-others and total-out-to-others, respectively, i.e., the total spillover that an individual in a specific market receives from individuals in another type of market and the total spillover of an individual in a specific market on individuals in another type of market. The TIFO and TOTO indices can be calculated using Eq (9) and Eq (8), respectively. (v) Sector spillover is used to measure the spillover from one market to another, which can be calculated using Eq (10).

Panel B of Table 1 further exhibits the cross-market (sector) spillover effect between EPU and ERV. The results are similar to those of Panel A. As the source of risk, ERV is found to have a strong spillover effect on EPU (171.81), accounting for 6.48% of the total spillover; the impact intensity of EPU on ERV is 81.43 (accounting for 3.07% of the total spillover). The sector spillover effect of ERV on EPU is approximately 2.1 times that of EPU on ERV, showing a prominent asymmetric two-way spillover effect between EPU and ERV. As the source of risk contagion, ERV has a large spillover effect on EPU; although EPU also has a spillover effect on ERV, such an effect is smaller.

### 3.2 Ranking of global systemically important markets

Based on the above analysis, the network spillover indices of EPU and ERV of the 21 countries are ranked. The top one-third of the markets (i.e., seven countries) are listed in Table 2. According to the “out” index, the ERV of the United States, Australia and Eurozone countries (Spain, Germany, Italy, France and Netherlands) is more risk contagious. In addition, the outward spillover effects of the EPU of developed countries (e.g., the United States) are greater; hence, the EPU of these countries is the main factor that causes economic policy adjustments and foreign exchange market shocks on a global level. Next, the “out” indices of EPU and ERV are added to obtain the total outward spillover index (i.e., total out) of each country. The results show that the top one-third total outward spillover effects are found in the United States, Australia and the five Eurozone countries.

**Table 2:** Ranking of global systemically important markets.

	Out			Cross-market spillover			
				From EPU to ERV		From ERV to EPU	
Ranking	ERV out	EPU out	Total out	EPU TOTO	ERV TIFO	ERV TOTO	EPU TIFO
**1**	Spain (120.20)	US (99.74)	US (214.49)	Netherlands (7.23)	Colombia (8.52)	Mexico (16.88)	Netherlands (16.42)
**2**	Germany (115.73)	Australia (63.60)	Australia (179.29)	US (6.74)	Mexico (7.35)	Colombia (14.16)	Mexico (12.07)
**3**	Australia (115.68)	Canada (60.92)	Germany (174.89)	Mexico (5.92)	Russia (6.93)	Japan (13.35)	Brazil (11.12)
**4**	Italy (115.34)	Germany (59.16)	France (171.18)	UK (5.23)	Japan (6.01)	Australia (10.85)	Russia (9.85)
**5**	France (114.92)	Korea (57.39)	Spain (164.44)	Germany (4.67)	India (5.54)	Spain (10.33)	Japan (9.74)
**6**	US (114.75)	France (56.26)	Netherlands (154.92)	Italy (4.63)	Greece (4.08)	Italy (9.29)	US (9.66)
**7**	Netherlands (111.16)	UK (50.64)	Italy (154.80)	Japan (4.42)	Brazil (4.01)	Germany (9.15)	Italy (9.31)

Note: (i) ERV represents exchange rate volatility, and EPU represents economic policy uncertainty. (ii) "Out" denotes the outward spillover index, which represents the total spillover of a single individual on all other individuals. The "out" index can be calculated using Eq (6). (iii) The total out index is obtained by adding the “out” indices of EPU and ERV. (iv) TIFO and TOTO are market-specific spillover indices, and the two denote total-in-from-others and total-out-to-others, respectively, i.e., the total spillover that an individual in a specific market receives from individuals in another type of market and the total spillover of an individual in a specific market on individuals in another type of market. The TIFO and TOTO indices can be calculated using Eq (9) and Eq (8), respectively.

In terms of the cross-market spillover of ERV on EPU, the TOTO index of the ERV shows that the ERV of both developing countries, such as Mexico and Colombia, and developed countries, such as Japan, Australia and the Eurozone countries, have a strong spillover effect on global EPU. The TIFO index of EPU reveals that the EPU of both developed and developing countries is susceptible to volatility in global foreign exchange markets. Among the top seven countries, four are developed countries and three are developing countries. Thus, a risk contagion path of “global ERV → global EPU” is uncovered.

In terms of the cross-market spillover of EPU on ERV, the TIFO index of the ERV demonstrates that the foreign exchange markets of developing countries are more vulnerable to shocks from global EPU. Specifically, the top three countries are all developing countries (namely, Colombia, Mexico and Russia), and five of the top seven countries are developing countries (namely, Colombia, Mexico, Russia, India and Brazil). This suggests that the foreign exchange markets of these developing countries are more vulnerable to shocks when adjustments occur in the global economic policy. To test whether the spillover effects of global EPU concentrate on the foreign exchange markets of developing countries, we further divide the sample into developing and developed country groups, and the average spillover effects between EPU and ERV of the two groups are calculated, with the results presented in Table 3. Given that the number of developing countries (7) is not equal to that of developed countries (14) in our sample, a simple sum of the spillover effects by group could not be used for a between-group comparison. To ensure the spillover effects are comparable across different groups, the means of the spillover effects in each group (sum of the spillover effects/the number of spillover relations in the group) is used to compare the spillover intensity. Table 3 shows that the spillover effects of EPU on ERV are indeed concentrated in developing countries. The average spillover intensity of developing countries’ EPU and developed countries’ EPU on the ERV of developing countries are both 0.27, which is significantly greater than their respective average spillover intensity on the ERV of developed countries (0.13 and 0.15, respectively). This suggests that changes in global EPU are a key reason for the volatility in the foreign exchange markets of developing countries because they are more susceptible to shocks from global economic risk. Thus, a risk contagion path of “global EPU → ERV in developing countries” is obtained.

**Table 3:** Average spillover effects between EPU and ERV in developed and developing countries.

From\To	EPU (developing countries)	EPU (developed countries)	ERV (developing countries)	ERV (developed countries)
**EPU (developing countries)**	1.53	1.67	0.27	0.13
**EPU (developed countries)**	1.89	2.56	0.27	0.15
**ERV (developing countries)**	0.50	0.39	3.17	2.14
**ERV (developed countries)**	0.37	0.37	3.10	4.80

Note: (i) ERV represents exchange rate volatility, and EPU represents economic policy uncertainty. (ii) This table uses the means of the spillover effects in each group (i.e., sum of spillover effects/the number of spillover relations in the group) to measure spillover intensity to ensure that spillover effects are comparable across different groups.

In addition, Table 3 also shows that the spillover effects of ERV on EPU are not concentrated in either developing or developed countries. The mean intensity of the spillover of the ERV of developing countries on their own EPU and the EPU of developed countries are 0.50 and 0.39, respectively, which are not significantly different. The mean intensity of the spillover of the ERV of developed countries on their own EPU and the EPU of developing countries are both 0.37. These findings further support the previous conclusion that the risk contagion path is “global ERV → global EPU.”

In summary, there is a prominent asymmetric spillover effect of ERV on EPU at the global level, and the spillover effect of EPU on ERV is mainly concentrated in the foreign exchange markets of developing countries. Therefore, the risk contagion between global EPU and ERV spreads along the path of “increasing global ERV → rising global EPU → further intensified volatility in the foreign exchange markets of developing countries”.

### 3.3 Dynamic evolution of the spillover effects between global EPU and ERV

**Fig 1:**
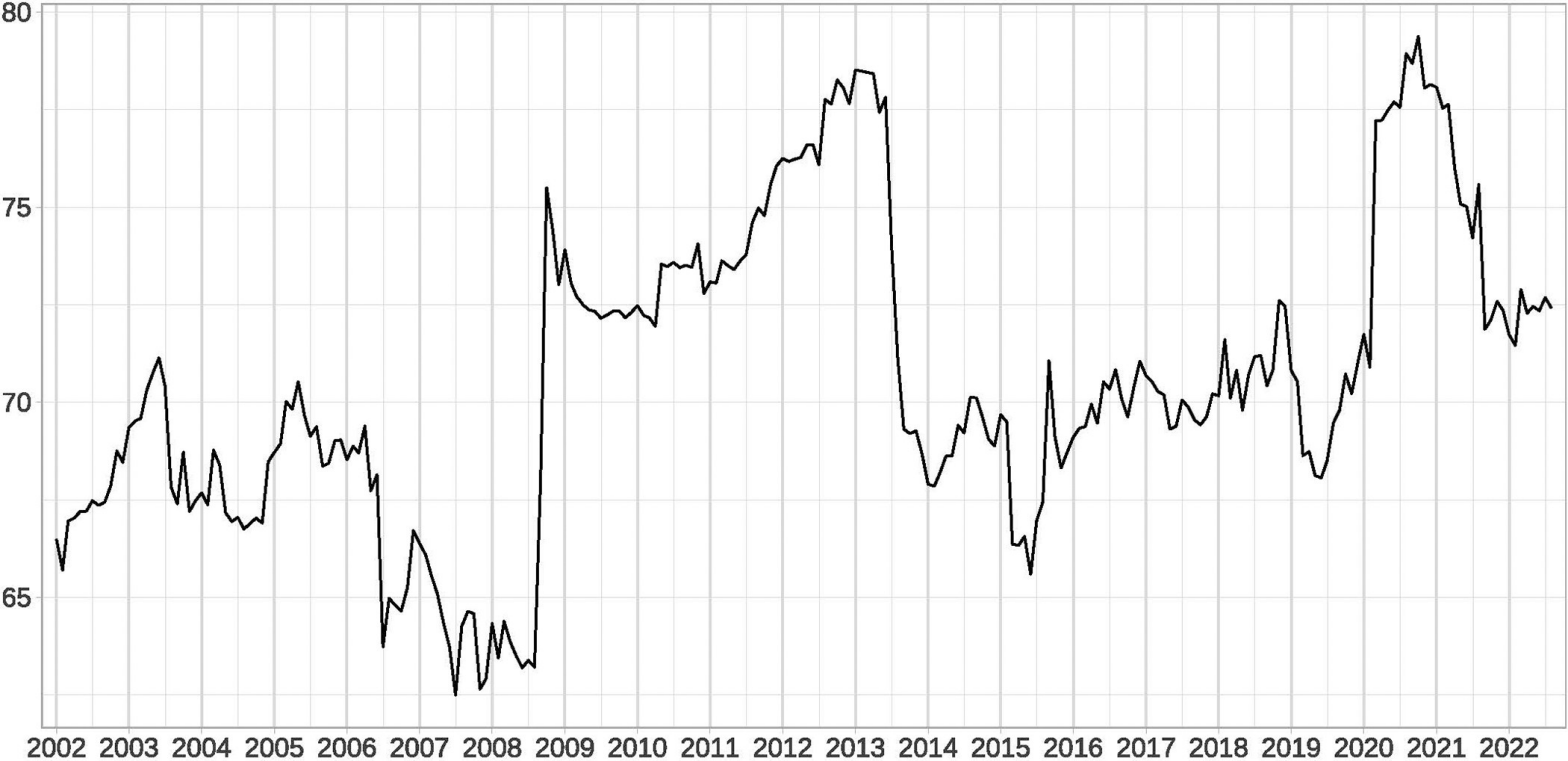
Time-varying characteristics of the total spillover of the network of global EPU and ERV. (i) This figure plots the total spillover between global EPU and ERV for a 60-month rolling window. (ii) Since we use a rolling window of 60 months and our sample starts in 1997, the starting year of this figure is 2002.

With 60 months as a rolling window, we use rolling spillover indices to measure the dynamic cross-border and cross-market spillover intensity between global EPU and ERV. Fig 1 shows the time plot of the total spillover of the network of global EPU and ERV. The total spillover increased significantly as an effect of extreme international events. Specifically, during the 2008–2013 crisis period (including the GFC and the European sovereign debt crisis), the total spillover rose drastically. Following 2014, the spillover intensity dropped to a similar level to that prior to the crisis. However, following the outbreak of the COVID-19 pandemic in early 2020, the total spillover increased rapidly and peaked at a level comparable to that seen during the 2008–2013 crisis in the second half of 2020. By 2021 and 2022, the level of total spillover begins to decline, although it still remains at a higher level than in the pre-pandemic period.

**Fig 2:**
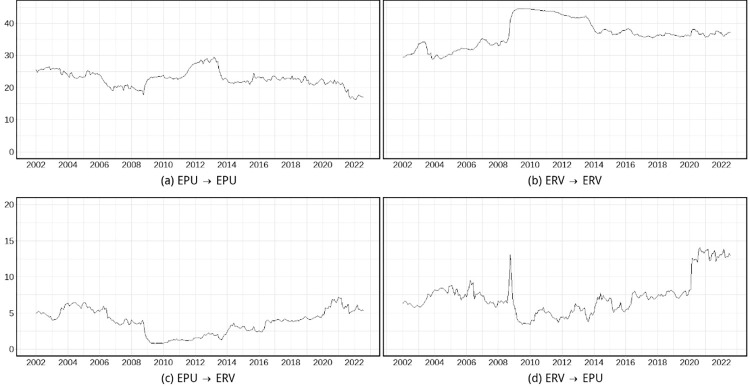
Time-varying characteristics of intra/inter-sector spillovers of global EPU and ERV. (i) This figure plots the intra/inter-sector spillovers of global EPU and ERV for a 60-month rolling window. (ii) Since we use a rolling window of 60 months and our sample starts in 1997, the starting year of this figure is 2002.

Fig 2 further decomposes the total spillover into intra-sector and inter-sector spillovers. The increase in the total spillover during the 2008–2013 crisis period was mainly caused by the increase in the respective intra-sector spillover in EPU and foreign exchange markets. The cross-border spillover intensity within EPU rose from approximately 20 prior to the crisis to a maximum of nearly 30 during the crisis. The cross-border spillover intensity of within foreign exchange markets rose from approximately 35 to a maximum of nearly 45. In addition, at the beginning of the 2008 GFC, the spillover effect of ERV on EPU manifested a brief rise, followed by a sharp decline.

In the post-crisis period, the intra-sector spillovers in both EPU and foreign exchange markets have declined; however, the cross-market spillover effect between the two has continued to rise. Moreover, a comparison between Fig 2C and 2D shows that the spillover of ERV on EPU has remained greater than that of EPU on ERV, indicating that the asymmetric feature also holds under the rolling window.

Following the outbreak of the COVID-19 pandemic in 2020, the total spillover has shown another drastic increase, which is primarily due to an increase in the spillover of ERV on EPU, followed by an increase in the spillover of EPU on ERV. While in the year of the pandemic outbreak (i.e., 2020), the level of intra-sector spillovers within the EPU and ERV sectors did not exhibit significant variations. By 2021 and 2022, the spillover intensity of ERV to EPU has continued to remain high, and the spillover intensity of EPU to ERV has decreased but still remain higher than its pre-pandemic level. However, the intra-sector spillovers within EPU have not risen after the pandemic but rather have fallen. This could be due to the weakened global economy and trade links caused by the pandemic, which inhibited the mutual influence of economic policy adjustments between countries.

### 3.4 Dynamic spillover network between global EPU and ERV

**Fig 3:**
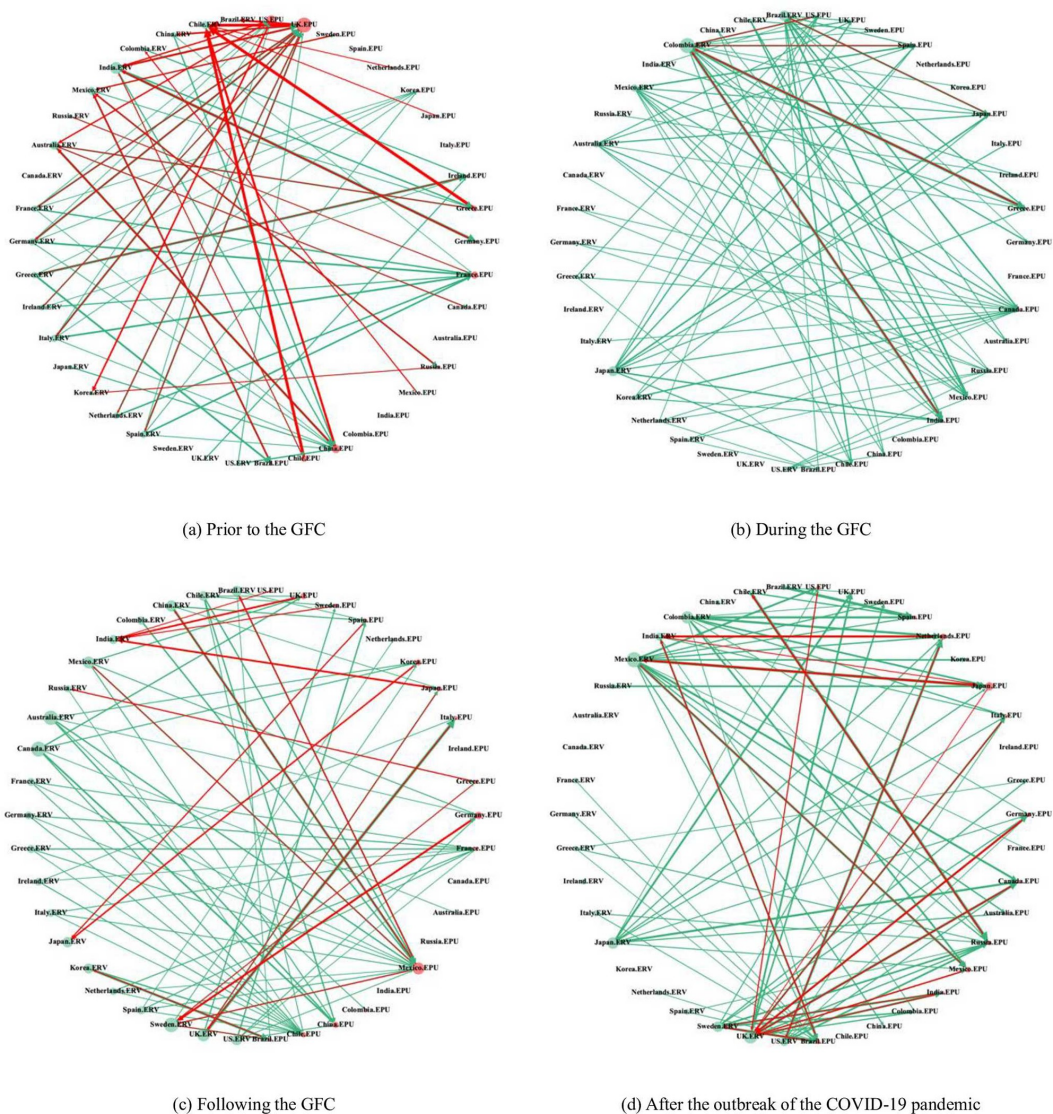
Cross-market spillover network between EPU and ERV in different periods. (i) X.EPU represents the EPU of country X, and X.ERV represents the exchange rate volatility of country X. (ii) The figure shows the top 10% quantile of the cross-market spillover relationships between EPU and ERV, where the green line represents the spillover of ERV on EPU, and the red line represents the spillover of EPU on ERV. (iii) The arrow represents the direction of the spillover; the thickness of the line represents the intensity of the spillover. (iv) (a), (b), (c) and (d) correspond to February 2003, March 2009, August 2015 and May 2020, respectively.

Based on the above analysis, we select four representative periods (prior to the GFC, during the GFC, following the GFC, and after the outbreak of the COVID-19 pandemic) to construct network connectedness diagrams to further investigate the dynamic changes in the structure of the spillover network between global EPU and ERV. Fig 3 reveals the noticeably asymmetric spillover relationship between EPU and ERV. The spillover of ERV on EPU (green line) is significantly greater than that of EPU on ERV (red line). In addition, the cross-market spillover between EPU and ERV is time-varying. In the post-GFC period, the cross-market spillover between EPU and ERV (particularly, the spillover of EPU on ERV) began to rise. After the outbreak of the COVID-19 pandemic in 2020, the cross-market spillover between EPU and ERV increased rapidly and substantially within a short period.

## 4. Conclusion

Using data during 1997–2022 from 21 major countries, this study constructs a spillover network between EPU and ERV to investigate the relationship of the cross-border and cross-market risk spillover effects between global EPU and foreign exchange markets. The results show a significant asymmetric bidirectional spillover effect between EPU and ERV, where the spillover effect of ERV on EPU is greater than that of EPU on ERV. Furthermore, the spillover of EPU on ERV is mainly concentrated in the foreign exchange markets of developing countries. These findings suggest that the spreading of risk contagion between EPU and ERV mainly follows the pathway of “increase in global ERV → rising global EPU → further intensified volatility in the foreign exchange markets of developing countries.” Thus, the foreign exchange markets of developing countries are more vulnerable to shocks from global economic risk. The dynamic analysis based on a 60-month rolling window reveals that the spillover intensity of the network of global EPU and ERV is time-varying. The cross-market spillover between EPU and ERV has continued to increase during the post-crisis period. In particular, following the 2020 outbreak of the COVID-19 pandemic, the total spillover intensity of the global network rose sharply to a point as high as that seen during the 2008–2013 crisis period, and it is largely due to the rapid and drastic increase in cross-market spillover between EPU and ERV.

Due to the complex risk spillover relationships between EPU and ERV in both cross-border and cross-market dimensions, our findings are of great significance to policymakers across the globe and may help them prevent potential risks. First, countries should improve the measurement, monitoring and early warning systems for shocks from global EPU. In particular, since the foreign exchange markets of developing countries are the main recipients of international risk contagion, they are most vulnerable to shocks from global economic risk. Therefore, developing countries should not only incorporate their domestic EPU indices but also EPU indices from other countries that have significant spillover to their foreign exchange markets into their forex risk prevention, monitoring and early warning system to maintain the stability of their foreign exchange markets. Second, on the basis of accurate identification of the characteristics of the risk spillover network, as well as the transmission structure between global EPU and ERV, countries should focus on controlling risks from countries of significant dependence. International policy coordination and cross-border supervision cooperation are suggested to accurately prevent and control potential risk spillover from abroad, effectively cutting off the risk transmission path and curbing the cross-border contagion and spread of overseas EPU and forex market risk. Third, while striving to ensure continuity, stability and transparency during the processes of economic policy formulation and revision, countries should further strengthen international policy coordination and establish an improved comprehensive and multi-level exchange and sharing platform for global economic policies to minimize the cross-border and cross-market contagion caused by information asymmetry. Lastly, since the spillover effects between global EPU and ERV tend to rise significantly under the shocks of extreme events, countries should pay close attention to extreme risk events such as the COVID-19 pandemic and actively prevent and control the effect of imported risk.

Some limitations exist in this study. First, since our EPU data are obtained from the database of EPU indices for major global countries constructed and published by Baker et al. [1] and the number of countries covered in the database is somewhat limited, we can only select EPU index data for a maximum of 21 countries as our sample, and some other important developed economies and emerging markets and developing economies are not included. In the future, if we can further obtain EPU index data for more countries, we would try to construct a global risk spillover network between EPU and ERV that includes more countries and then more comprehensively analyze the cross-border and cross-market spillovers between global EPU and ERV as well as their risk transmission pathways. Second, this study focuses on the risk spillover network between global EPU and ERV, and future studies could consider incorporating more financial markets (e.g., stock markets) into the network, which may lead to new insightful findings.
